# Quantification of histone modification ChIP-seq enrichment for data mining and machine learning applications

**DOI:** 10.1186/1756-0500-4-288

**Published:** 2011-08-11

**Authors:** Stephen A Hoang, Xiaojiang Xu, Stefan Bekiranov

**Affiliations:** 1 Department of Biochemistry and Molecular Genetics, University of Virginia Health System, Charlottesville, Virginia, USA

## Abstract

**Background:**

The advent of ChIP-seq technology has made the investigation of epigenetic regulatory networks a computationally tractable problem. Several groups have applied statistical computing methods to ChIP-seq datasets to gain insight into the epigenetic regulation of transcription. However, methods for estimating enrichment levels in ChIP-seq data for these computational studies are understudied and variable. Since the conclusions drawn from these data mining and machine learning applications strongly depend on the enrichment level inputs, a comparison of estimation methods with respect to the performance of statistical models should be made.

**Results:**

Various methods were used to estimate the gene-wise ChIP-seq enrichment levels for 20 histone methylations and the histone variant H2A.Z. The Multivariate Adaptive Regression Splines (MARS) algorithm was applied for each estimation method using the estimation of enrichment levels as predictors and gene expression levels as responses. The methods used to estimate enrichment levels included tag counting and model-based methods that were applied to whole genes and specific gene regions. These methods were also applied to various sizes of estimation windows. The MARS model performance was assessed with the Generalized Cross-Validation Score (GCV). We determined that model-based methods of enrichment estimation that spatially weight enrichment based on average patterns provided an improvement over tag counting methods. Also, methods that included information across the entire gene body provided improvement over methods that focus on a specific sub-region of the gene (e.g., the 5' or 3' region).

**Conclusion:**

The performance of data mining and machine learning methods when applied to histone modification ChIP-seq data can be improved by using data across the entire gene body, and incorporating the spatial distribution of enrichment. Refinement of enrichment estimation ultimately improved accuracy of model predictions.

## Background

Recent advances in high-throughput DNA sequencing technology have facilitated the generation of vast amounts of epigenomic ChIP-seq data. The availability of these datasets has provided the opportunity to utilize the power of statistical computing to model epigenomic networks. Unlike conventional biochemical approaches, the application of machine learning and data mining techniques to ChIP-seq data is capable of providing a broad, network-level view of the epigenetic regulatory system. These strategies can provide insights into mechanisms of genomic control, such as the so-called "histone code [1-3]," by facilitating an integrated analysis of the many histone posttranslational modifications (PTMs) that have been described, as well as other epigenetic chromatin modifications. The histone code is a particularly attractive problem for computational applications, since it has become apparent that histone PTMs are regulated in a network fashion and are deposited combinatorially [4-6]. However, a thorough study of the gene-biased quantification of ChIP-seq enrichment for the application of data mining and machine learning techniques has not yet been done.

Several groups have applied various data mining and machine learning techniques to epigenomic ChIP-seq data in a gene-biased fashion, including Bayesian networks [7,8], support vector machines and regression [8], linear [6,8,9] and nonlinear regressions [6]. These models have focused on histone modifications due in part to their known role in transcriptional regulation [10-12] and the availability of rich datasets exhibiting a wide variety of types of histone modifications [10]. These models have been built using individual genes as observations, ChIP-seq enrichments as predictors, and gene expression as responses in the case of supervised learning methods. In these models the predictors are histone modification/variant ChIP-seq enrichment levels for individual genes, so model quality is highly dependent on the accuracy of enrichment estimation. Since ChIP-seq enrichment levels are strongly dependent on genomic coordinate, providing a gene-wise estimate of ChIP-seq enrichment that accurately captures the relevant enrichment information across all genes--which vary in length over four orders of magnitude--is a challenging task.

The most straightforward way to estimate per-gene ChIP-seq enrichment is to simply count the number of sequence reads associated with a given gene. Indeed, counting the number of reads in the promoter region of each gene was an approach taken in some previous studies [7,9]; however, this method has several limitations. First, tag-counting methods equally weight every position within the counting window, and thus ignore the spatial component of the enrichment data. Second, not every histone modification is 5' biased. Several modifications have greater enrichment into the body of genes, such as H3K36me3 [10,12,13]. This modification highlights the pitfalls associated with 5' biased enrichment estimation. It has greater enrichment in the bodies of genes, is indeed 3' biased, and has a proclivity for enrichment in exons [13,14]. H3K36me3 also has a strong correlation with transcriptional elongation as determined by various biochemical studies [10,14-16]. It is worth noting that a machine learning study by Yu et al. used a 5' tag counting method and found little correlation between H3K36me3 enrichment and gene expression [7]. The consequence for models that use 5' proximal enrichment estimation methods is that the effects of histone modifications with gene body or 3' biased enrichment are underestimated or greatly obscured.

Histone methylations tend to have unique average spatial deposition patterns [10]. For example, in contrast to H3K36me3, H3K4me3 has high enrichment around the transcription start site, with depletion in the nucleosome-free region. Some modifications seem to be deposited in a specific genic region with respect to the absolute positions of nucleosomes relative to gene boundaries, while the patterns of others seem to scale with gene length. These effects can be attributed, at least in part, to the recruitment of histone methyltransferases that are dependent on the phosphorylation state of the C-terminal domain of Pol II before and during the elongation process [13,16-18]. Estimating ChIP-seq enrichment is complicated by the different modes of histone PTM deposition coupled with the wide variability in gene lengths. Part of the information content associated with a given histone modification is encoded within the spatial distribution of the enrichment data, and so it should also be considered when estimating enrichment levels.

The selection of a genomic window used for the calculation of enrichment levels is important in capturing relevant enrichment data, since enrichment information may be 5' or 3' proximal, intergenic, or intragenic [8]. Choosing a window size that extends too far outside of gene boundaries may incorporate data from neighboring regions, and selecting a window size that is too small may exclude useful data. The goal in window selection is to maximize useful information content and minimize the incorporation of noise, while being generalizable across a variety of marks.

Since the quality of a statistical model is largely dependent on the quality of the observation data used to build it, refining enrichment estimation methods is important for future statistical analyses of ChIP-seq data. To resolve some of the issues involved with enrichment estimation, we compared the performance of models built using a ChIP-seq dataset of histone methylations/variant in CD4^+ ^T-cells generated by Barski et al. [10]. This dataset has been used in several other machine learning and data mining studies [6,7,9]. We applied the Multivariate Adaptive Regression Splines (MARS) algorithm [19,20] to build nonlinear regression models using enrichment levels of 20 histone lysine and arginine methylations plus histone variant H2A.Z. Given that gene expression levels have been shown to be highly dependent on histone modification levels, we used the Generalized Cross-Validation (GCV) [19] and R^2 ^metrics to assess the quality of MARS model fits and rank enrichment estimation methods.

Several different strategies were employed for estimating gene-wise enrichment levels, including tag counting and model-based approaches, which use average enrichment patterns to spatially weight enrichment of individual genes in a set of genomic windows. We also investigated the selection of window sizes for our gene-wise enrichment estimation methods. By comparing models using GCV and R^2 ^values, we demonstrate that the performance of regression models using histone modification enrichment levels as predictors can be greatly affected by the chosen enrichment estimation method. We conclude that methods that incorporate the spatial distribution of ChIP-seq enrichment offer an improvement in a regression fit over tag counting methods. We also observe that whole-gene estimation windows produce superior results relative to estimations restricted to specific genic regions. Indeed, incorporating data across the entire body of the gene was the most important factor in improving the fit of our models. These improvements of gene-wise ChIP-seq enrichment estimation can improve the sensitivity and specificity of the predictions derived from data mining and machine learning models.

## Methods

### Gene selection

A list of gene transcript annotations was downloaded from the NCBI36 *Homo sapiens *database, Ensembl 54 (June 24, 2009), which was then filtered to only include transcripts that had Ensembl, UCSC, and RefSeq IDs. Genes that did not have corresponding expression data associated with them were removed from the list. Many of the transcripts within this list contain multiple annotated start and stop sites. Using the same procedure described by Xu et al. [6], we select a single Transcription Start Site (TSS) and Transcription End Site (TES) for each gene.

Some of the enrichment estimation methods described in this study calculate enrichment within a window around the TSSs and TESs, the largest of which we employed was ±3000 bp around each site. To avoid overlap between windows where the enrichment estimate was a combination of estimates from both ends of the genes, the gene list was further filtered to only include transcripts of length 6002 bp or greater. Although not all enrichment estimation strategies have this limitation, the filtered list was used for each enrichment estimation method to allow a fair comparison of the final models. After implementing each of the aforementioned filters, the final gene list totaled 9882 genes.

### Tag Repeat Filter

PCR sequence artifacts or phenomena inherent to the sequencing technology may cause repeat sequences to be produced. These artifacts manifest as large numbers of tags that map to precisely the same genomic coordinates. With the exception of H3K79 methylations, maximum repeats ranged from 231 for H3K4me2 to 4231 for H3K9me3. H3K79me1/2/3 had far fewer repeats with maximums of 23, 26, and 42, respectively. We identified these tag "pile-ups" by searching for multiple tags that mapped to the genome with precisely the same start and stop coordinates (or for differing tag lengths, within the margin of the difference in length). A cutoff of 75 repeats was chosen empirically for the modification H3K4me3 (max repeats = 1166) to filter repeat artifacts from H3K4me3 data. We assumed that the typical number of tags in these piles for a given mark crudely scaled with the total number of tags. Thus, the cutoff was scaled for other modifications by the total tag count relative to H3K4me3, and ranged from 21 (H3K79me2) to 75 (H3K4me3). H3K4me3 was chosen to determine the cutoff because it had the largest total tag count, and its tendency to form large localized peaks relative to the other modifications. This helped ensure that our cutoff was not overly stringent and was only sensitive to extreme outliers.

Using this filtering scheme H3K79me1/2/3 had data removal percentages of 0%, 0.001%, and 0.004%, respectively. H4K20me3, which had relatively large numbers of reads that mapped to repeat sequences, had 5% of all data removed. All other marks ranged from 0.01% (H3K4me1) to 0.5% (H3K9me3) of data removed. Thus, we removed extreme outliers while minimally affecting the overall data set.

### Tag counting

Tag counting is the summation of ChIP-seq reads within a genomic window. Any part of a read falling within a window was included in our tag counts. Following previous studies using tag counting methods, tag count enrichments were calculated in ±500 bp, ±1000 bp, ±2000 bp, and ±3000 bp windows relative to both the annotated Transcription Start Sites (TSS) and the Transcription End Sites (TES). While evaluating which genic sub-regions to include in tag counting methods, we assessed how the inclusion of tag counts within exons as a genic sub-region category improved model performance and found their contribution was negligible (Additional file 1). We therefore did not consider tag counts solely within exons further. For clarity, exons were not excluded from other counting methods. Another set (one for each window size) of gene-wise count-based enrichment estimates were produced by summing the TSS counts with the TES counts multiplied by a scaling factor for each of the 21 histone marks:(1)

Where *j *is the gene, *k *is the modification type, *E *is enrichment estimate, *C *is the tag count in the window, and α is a scaling factor. The purpose of the scaling factor, *α*, is to effectively weight the contribution of the 5' and 3' ends with respect to gene expression. The scaling factor was calculated for each modification by optimizing the absolute value of the correlation between the sum of the two tag count values and gene expression:(2)

where *Y *is gene expression level. The correlation was optimized numerically with respect to *α*, for each modification type, *k*.

A set of whole-gene tag count enrichments was calculated within a window defined by the gene boundaries plus flanking intergenic regions immediately adjacent to the annotated gene boundaries. Counts were normalized by dividing by the length of the counting window. Sets of normalized counts were calculated for the gene bodies plus 0 bp, 500 bp, 1000 bp, 2000 bp, and 3000 bp overhangs up and downstream of the gene boundaries.

### Iterative model-based enrichment estimation

Using a strategy similar to the one described by Xu et al. [6], we create a "template" *t_ik _*for each mark *k*. The template is the normalized average enrichment profile for a given mark, within a window relative to gene coordinates, *i*:(3)

where *c_ijk _*is the enrichment of a mark *k *for a gene *j *at genomic coordinate *i *and *N *is the total number of genes. Templates were normalized by a constant such that:(4)

where  is the normalized template, and *N *is the number of bins. We assume that the enrichment profile of a given gene can be approximated by a template *t_ik _*multiplied by an enrichment level estimate *X_jk _*of a mark *k *for a given gene *j*. The least squares difference *Q_jk _*between the estimated enrichment profile *X_jk_t_ik _*and the actual data is given by:(5)

By minimizing *Q_jk _*with respect to the enrichment estimate *X_jk _*and applying the normalization constraint given by equation 4 we arrive at the following enrichment estimate equation:(6)

In addition to using a non-weighted average template as shown in equation 3, we minimized *Q_jk _*with respect to the template *t_ik _*to arrive at the following enrichment estimate weighted tag count template equation:(7)

Equations 6 and 7 can be solved iteratively, subject to the template normalization constraint given by equation 4. An iterative solution of these equations minimizes the least squares difference between the modeled enrichment data  and the actual data *c_ijk_*. In the case of the iterative solution, the template is the enrichment estimate weighted average tag count across genomic coordinate, *i*. The value of *X_jk _*is ultimately a weighted average of enrichment across a genomic window, providing a single-value estimate of enrichment that incorporates information from the spatial distribution of the enrichment data. For our calculations the iterative process continued until the average difference between the n^th ^and the (n+1)^th ^set of enrichment estimations converged to less than 5% of the n^th ^set values.

Using this iterative model-based strategy, enrichment levels were estimated around both the TSS and TES in ±500 bp, ±1000 bp, ±2000 bp, and ±3000 bp windows, with single base pair resolution (i.e., *i *corresponds to a single base pair in the window). Enrichment estimates were also made with templates consisting of the TSS and TES windows combined (calculated as a single template) using the same four window sizes. In summary, a set of 5', 3', and 5'+3' enrichment estimations were made for each of the window sizes.

In another set of enrichment estimates, genes were scaled to correspond to a fixed number of bins. The scaling procedure described by Xu et al. [6] was used with bin number equal to 33,346--the median gene length in the filtered gene list. The template procedure was applied to the scaled genes plus an intergenic overhang of 0 bp, 500 bp, 1000 bp, 2000 bp, and 3000 bp beyond the TSS and TES. The resolution of the genes is equal to gene length divided by bin number, while the overhang regions have base-pair resolution.

### Non-iterative model-based enrichment estimate

The process of iteratively solving equations 4, 6, and 7 is computationally expensive. A non-iterative enrichment estimation can be made with equation 6 using the non-weighted average template shown in equations 3 and 4. To examine the trade off between computational efficiency and template optimization, we produced one set of enrichment estimates calculated non-iteratively for every set calculated using the iterative method.

### Evaluation of template models

Following Xu et al. [6], we used the coefficient of variation of the root mean square deviation CV(RMSD) to evaluate the fit of our templates:(8)

where n is the number of indices in the template. This metric was used to compare the fit of iterative and non-iterative template models.

### MARS model construction and evaluation

MARS models were built with each set of enrichment estimations (51 in total) using the *earth *package in R http://cran.r-project.org/web/packages/earth/index.html. Following Xu et al., each model was allowed terms with up to 3 degrees of interaction. The quality of each model was evaluated using R^2 ^values and GCV scores. The GCV score evaluates the fit of the model while penalizing model complexity, whereas the R^2 ^only considers the fit of the model to the data. A description of the MARS algorithm and GCV scores can be found in the 'Methods' section of Xu et al. [6].

## Results and discussion

### Overview of model construction

**Figure 1:**
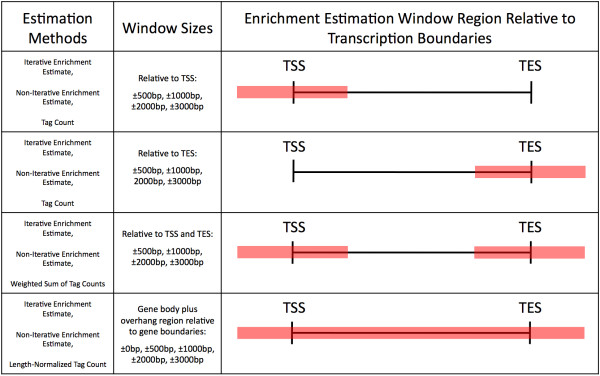
**Illustration of enrichment estimation methods**. Summary of the methods used to make single-value estimates of gene-wise ChIP-seq enrichment. The first column lists the enrichment estimation methods. The second column lists the window sizes for which each method is applied. The last column shows a graphical representation of the estimation region for each method/window size combination relative to the transcription start sites (TSS) and transcription end sites (TES) of genes.

A total of 51 enrichment level estimates were made for 21 marks for 9882 Ensembl genes, corresponding to 51 different MARS models. Figure 1 shows a summary of each enrichment estimation method. The responses of the models are gene expression data in CD4^+ ^T cells gathered from the SymAtlas database [21]. In cases where multiple Affymetrix probe sets interrogated a single gene, additional observations were included in the model corresponding to each independent expression measurement with redundant enrichment data, resulting in 15,148 observations and 21 predictors per model.

### Template model error analysis

To assess the fit of our template-based enrichment models to the enrichment data we used the CV(RMSD), as described in the Methods section. The CV(RMSD) was calculated and averaged for all genes above the 95^th ^percentile in enrichment estimations. Table 1 shows the CV(RMSD) for whole gene templates plus a 2000 bp intergenic overhang, for both non-iterative and iterative methods. In 13 of the 21 marks the iterative procedure improved the CV(RMSD); however, the iterative enrichment model performs more poorly than the corresponding non-iterative model for 8 marks.

**Table 1:** CV(RMSD) for whole gene templates plus a 2000 bp intergenic overhang

Mark	CV(RMSD)	
	Non-Iterative	Iterative
H2AZ	3.382	4.442

H2BK5me1	1.940	1.957

H3K27me1	1.973	1.970

H3K27me2	2.960	2.957

H3K27me3	2.599	2.601

H3K36me1	2.919	2.913

H3K36me3	1.793	1.838

H3K4me1	2.007	1.943

H3K4me2	2.366	2.310

H3K4me3	2.955	3.189

H3K79me1	1.812	1.806

H3K79me2	1.820	1.802

H3K79me3	1.798	1.795

H3K9me1	1.861	1.810

H3K9me2	3.046	3.055

H3K9me3	4.114	4.550

H3R2me1	2.524	2.521

H3R2me2	3.581	3.575

H4K20me1	1.497	1.489

H4K20me3	5.871	8.112

H4R3me2	3.175	3.174

The CV(RMSD) shows the fit of template models to the enrichment data. The first column shows the mark. The second column shows the CV(RMSD) for the non-iterative template. The third column shows the CV(RMSD) for the iterative template. The CV(RMSD) is improved (lowered) by the iterative template over the non-iterative template in 13 of the 21 marks.

The iterative and non-iterative H4K20me3 template models had the worst CV(RMSD)s (8.11 and 5.87, respectively). Moreover, the iterative template performed much more poorly than the non-iterative. In this case, H4K20me3 is highly enriched in members of the zinc-finger (ZNF) gene family, and at low levels with a different enrichment profile across the genes in the rest of the genome [10,22]. Thus, for H4K20me3, there are at least two classes of enrichment profiles across genes. The iterative template is weighted by enrichment, and hence biased toward the ZNF genes. Thus it yields a poor CV(RMSD) for the majority of genes in the genome that have a different profile and have relatively low levels of H4K20me3 across their bodies. One way of resolving this problem is to apply clustering analysis to the H4K20me3 enrichment profiles across genes and identify the two or three dominant deposition profiles and apply the appropriate template to each subset of genes. Nevertheless, the iterative template method required significantly more computational resources than the non-iterative method and only marginal improvements in the CV(RMSD) of 13 of the 21 marks, and in MARS model performance (as discussed in the "Enrichment estimation and model performance" section). This suggests that the non-iterative template approach may be preferable to the iterative enrichment estimation method for many applications.

### Enrichment estimation and model performance

**Figure 2:**
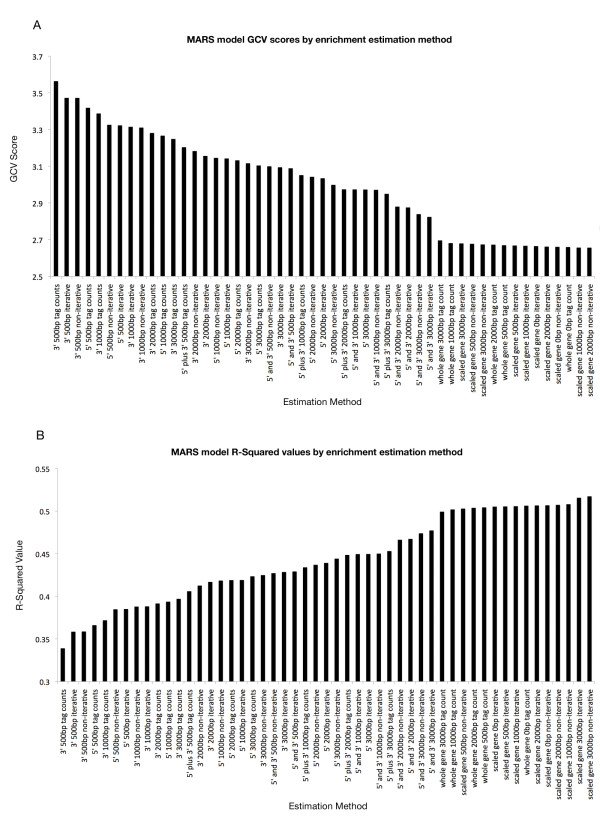
**Comparison of enrichment estimation methods by MARS model statistics**. Plots of (A) GCV and (B) R-squared values for MARS models built with each enrichment estimation method. GCV scores are sorted in descending order; small GCV scores are indicative of superior model fit. R-squared values are sorted in ascending order; large R-squared values are indicative of superior model fit. Models based on whole-gene enrichment estimates group together as the best models by both metrics.

We found a clear trend in model performance with respect to the enrichment estimation procedure used to calculate the model predictors. GCV scores range from 2.656 to 3.564 and R^2 ^values range from 0.517 to 0.339 across the 51 models. Figure 2 contains a summary of all models and their statistics. As expected, 3' estimates using small estimation windows yielded models with the poorest performance. Except for the whole gene estimates with no intergenic overhang, for equal window sizes, models based on tag counting estimates were always outperformed by either iterative or non-iterative template-based estimates, as measured by GCV score. With the exception of 2 (whole gene tag counts with 0 and 500 bp intergenic overhangs) out of the 17 tag count-based models, both the iterative and non-iterative template-based models outperformed the tag count-based models for the same window size. Models based on whole-gene estimates outperformed all other models.

The iterative enrichment estimation method was intended to improve the fit of the template to the data; however, this does not mean that the estimated enrichment level produces a final MARS model with a better fit. Indeed, we found this to be true in our models. Of the 17 pairs of iterative and non-iterative template-based enrichment estimations, 10 produced models in which the iterative method was superior, and 7 in which the non-iterative method was superior. However, both methods produced models with similar statistics (Figure 1). A possible explanation for this result is that the iterative method yields a template that is an estimation-weighted average of enrichment across genomic coordinates. Thus, genes with large outlier enrichment values for a given mark could be driving the shape of the iterative template. For H4K20me3, which produced the poorest CV(RMSD)s and a largest increase in CV(RMSD) in the iterative estimate relative to the non-iterative estimate, outliers did drive poor performance. As previously discussed ZNF repeats are highly enriched for this mark while most non-ZNF genes have an extremely modest enrichment. The genes that had the largest absolute deviation between iterative and non-iterative enrichment estimates were indeed ZNF genes. This suggests that datasets with extreme outliers may be poorly represented by the iterative enrichment estimate. Incorporating robust estimation procedures (e.g., trim mean) into template and enrichment estimation calculations may improve the results of the iterative enrichment estimation method.

Selection of window size was also a factor in model performance. For the window sizes considered, larger window sizes always yielded improved model fits for any 5' and/or 3'-focused method. Not surprisingly, an increase in the amount of data used to calculate the predictors generally improved the performance of the models. However, this does not hold true for the whole-gene tag counting and scaled-gene methods. For these methods, the size of the overhang region is a relatively small fraction of the total genomic coverage used in the per-gene enrichment estimation.

The estimate method with the best performance based on GCV score was the whole-gene, non-iterative, template-based method with a 2000 bp intergenic overhang, which achieved a GCV score of 2.656. The whole-gene method that received the poorest GCV score was the tag count with 3000 bp intergenic overhangs, which had a GCV score of 2.696. The difference in model GCV score between the best and worst whole-gene enrichment estimation methods was only 0.04, corresponding to 1.5% difference in GCV score; the associated p-value < 0.001. Significance was assessed by randomly permuting mark amplitudes with respect to genes. MARS was then applied to the randomly permuted data and GVC scores were calculated for the best and worst whole-gene enrichment estimation method as well as the percent difference in GCV score. A null distribution and corresponding p-value were calculated by repeating this procedure 1000 times. The worst whole-gene estimation method had a GCV score, which was 0.129 (4.6%) below that of the best method based on specific genic regions (5'+3' iterative template with 3000 bp intergenic overhang). The associated p-value < 0.001 based on the same random permutation procedure described above. This suggests that the most important factor when estimating gene-wise ChIP-seq enrichment is the inclusion of data across the entire length of gene bodies. Additionally, unlike the methods based on localized regions, the whole-gene methods do not show a strong correlation between model performance and window size; further suggesting that the enrichment data in the body of the gene contains the majority of the information content for a given gene.

The Spearman correlation between the iterative and non-iterative template-based (2000 bp intergenic overhang) enrichment estimates was 0.994 or better across all marks. As expected, the largest deviations between the methods were in estimates of H4K20me3 in the ZNF genes. Correlations of enrichment estimations between whole-gene tag counting and template-based methods (2000 bp intergenic overhang) had a median value of 0.983, and exceeded 0.925 for all marks except for H2A.Z and H3K4me3. The correlations between the tag counting method and the iterative and non-iterative methods for H2A.Z were 0.659 and 0.675 respectively, and 0.775 and 0.771 for H3K4me3. These relatively low correlations can be attributed to the fact that on average these two marks have extremely high enrichment within a few hundred base pairs of the TSS, which rapidly falls to nearly zero beyond 2000 bp into the gene body. No other marks show such a dramatic difference between the gene body and TSS region. For extremely large genes, this means an underestimation of the enrichment using the length-normalized tag count. Indeed, many of the largest deviations between the estimation methods for these marks were for genes that were on the mega-base scale in length (Additional file 2). Large deviations also occurred when few tags were observed within the estimation window. In these cases, differences between enrichment estimation methods can be attributed to coordinate-dependent differences in weighting. In some cases of 5' proximal marks, genes that were not enriched for the mark were flanked by genes that were (Additional file 3). The 5' enrichment of the neighbor would sometimes bleed into the 3' region of the non-enriched gene, causing a large enrichment estimate using the tag counting method relative to the template-based methods. Since the template-based methods are a weighting scheme based on the average enrichment pattern, the intruding enrichment is down-weighted. The template-based methods are subsequently able to deconvolve enrichment signals of genes that are close neighbors, and therefore represent an advantage of these methods over tag counting.

The accuracy and precision of amplitude estimation for all of the methods considered could be improved by subtracting background read levels and applying appropriate noise filtering. High throughput sequence analysis of input DNA samples revealed that chromatin structure affects shearing and other aspects of ChIP sample preparation, and hence introduces biases in ChIP-seq data [23]. This together with sequence dependent biases coming from PCR amplification of ChIP samples argues for methods that assume an inhomogeneous background. One approach would be to use input DNA or other control samples to estimate inhomogeneous background levels; however, an accurate method, which performs this analysis remains to be developed. Indeed a recent comparison of ChIP-chip and ChIP-seq data [24] showed that using Input-seq data as background from an *unmatched *sample can remove GC-content biases better than use of a matched Input-seq sample. Thus, accurate background estimation and subtraction is still an area of active research. One ChIP-seq peak finding method, SICER [25], which is designed to identify significantly enriched domains in histone modification data can also be applied as a background noise filter. SICER performs the filtering based on significance. The genome is segmented into windows and those that are not members of significantly enriched islands are filtered out (i.e., set to zero). However, a significance-based filtering approach is not ideal for amplitude estimation and statistical learning applications because accurate estimates of even low, albeit insignificant, enrichments are important. High frequency noise could be removed by applying low pass filters using wavelets. Indeed, wavelet analysis has been applied to genomic tiling array ChIP-chip data to denoise the data [26] and could be generalized for ChIP-seq noise filtering.

### Enrichment profile and gene length

**Figure 3:**
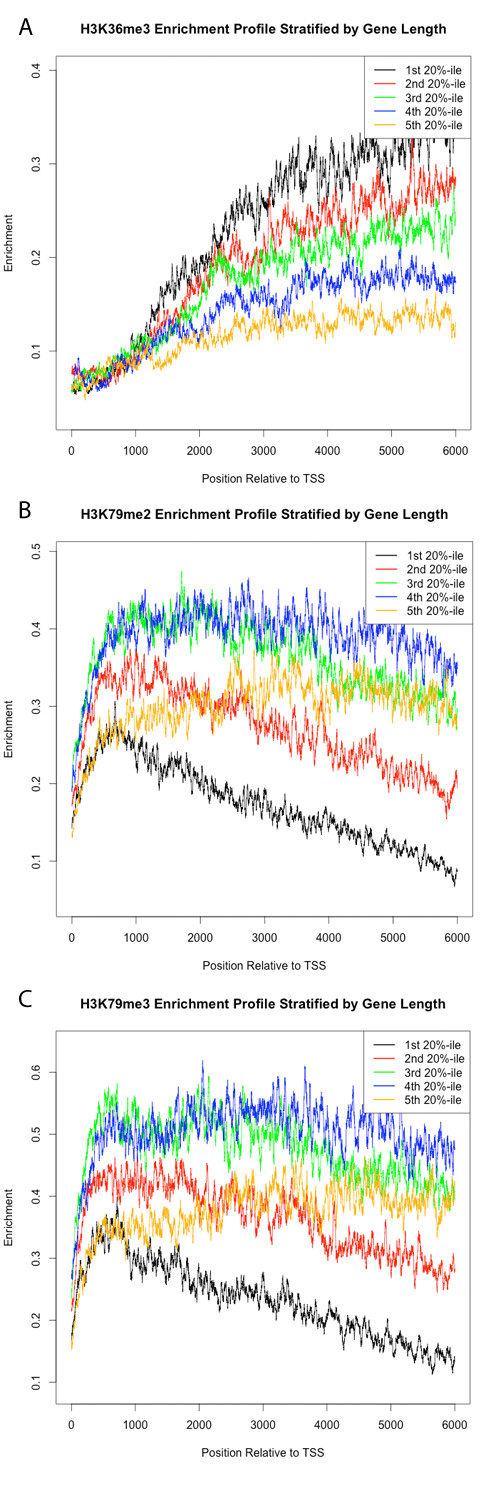
**Average histone modification enrichments stratified by gene length**. Plots of average enrichment profiles from the transcription start site to 6000 bp into the gene body for H3K36me3 (A), H3K79me2 (B), and H3K79me3 (C), stratified by quintiles of gene length. The variability in slope for each of these marks suggests that the enrichment pattern for each of these marks scale with gene length. For example, for the smallest 20%-ile of genes, H3K36me3 enrichment rapidly rises from the TSS to 6000 bp into the gene body; however, for each successive 20%-ile of increasing gene length, the rate of increase in enrichment is diminished for the same region.

The superlative performance of the scaled-gene enrichment estimation methods was unexpected considering many of the histone modifications in this study appear to have TSS-focused enrichment [10]. It was initially unclear as to whether scaling genes to calculate the enrichment template was appropriate, considering that these modifications are physically deposited on the tails of histones which make up nucleosomes that occupy ~146 bp of DNA. To determine whether a given mark is deposited with respect to the absolute position of nucleosomes or if it scales with gene length, we plotted normalized enrichment profiles of every mark from the TSS to 6000 bp into the gene body, stratified by quintiles of gene length (Figure 3, Additional file 4). Three marks displayed an enrichment pattern that distinctly scaled with gene length: H3K36me3 and H3K79me2,3 (Figure 3). Based on the presence of marks that scale with gene length and those that do not, we hypothesized that a template-based procedure based on absolute position of nucleosomes with the largest window size (i.e., 5'+3' template, 3000 bp window) would yield the best model. Such a model would accurately incorporate data that is based on absolute position of nucleosomes, and also capture the largest genomic region to incorporate the maximum amount of data from marks that scale with gene length. Despite this, and the fact that most of the marks do not appear to scale significantly with gene length (Additional file 4), the estimates based on scaled genes produced models with superior performance.

To determine if H3K36me3 and H3K79me2,3--all strongly associated with gene activation--were driving the superior performance of the scaled-gene models, we rebuilt all 51 MARS models without these predictors (data not shown). Surprisingly, the scaled gene method with no intergenic overhang yielded the best model, though the 2^nd ^and 3^rd ^best models were based on whole-gene tag counts. This suggests that although for many marks the scaled template is less representative of the deposition pattern of very large and very small genes, the scaled template strategy offers good performance even on marks whose enrichment profiles do not appear to scale significantly with gene length.

### Regulatory information embedded in spatial deposition patterns

Interestingly, the slopes of the enrichment profiles of the three marks that scale appear to be approximately similar across gene lengths from the TSS to approximately 1 kb into the gene body. Beyond approximately 1 kb into the gene body the slopes of the enrichment profiles begin to differ dramatically. For example, for the shortest 20%-ile of genes, average H3K79me3 enrichment rapidly decreases beyond 1 kb into the gene body. For the longest 20%-ile of genes the enrichment profile has a steady, positive slope for the same genomic window, which is about 1 kb to 6 kb into the gene body. However, from the TSS to approximately 1 kb into the gene body the enrichment profiles of these extreme length groups are nearly identical. This suggest that for these scaling marks, there is a region near the TSS, which is approximately 1 kb in size, where these modifications are deposited in a length-independent manner, but beyond which the modifications are deposited in a length-dependent fashion.

Of the non-scaling marks, all but four of the modifications in this study show differences in absolute enrichment levels across gene lengths (Additional file 4). Those that do not are H3K27me2, H3R2me2, H4R3me2, and H4K20me3. The greatest differences appear in the longest 20%-ile, which has relatively low enrichment for marks that are explicitly known to be associated with gene activation, and relatively high enrichment for genes associated with gene repression, suggesting a global reduction of transcription in large genes relative to small genes. Indeed, we find that the genes in the largest 20%-ile of gene length show significantly lower gene expression than other genes (Additional file 5). For methods that did not use the whole gene to arrive at enrichment estimates, we rebuilt models with gene length included as a predictor to determine if the superior performance of the whole-gene estimation methods were driven by the gene length bias. The best performing model of this set was the 5'+3' non-iterative template with a 3000 bp window, which had a GCV score of 2.831. The best model based on estimates in a specific genic region and without gene length as a predictor had a GCV score of 2.824. The lack of improvement after including gene length as a predictor suggests that the performance of the whole-gene enrichment estimation methods was not driven by the gene length bias.

In addition to revealing information about transcriptional regulation, templates and enrichment estimates may also provide information on co-regulation of PTMs. For example, H4K20me1 and H3K9me1 are known to be preferentially deposited on the same nucleosomes *in vivo *[27]. The correlation (Spearman) between the templates of these two marks was 0.716, and the correlation between their enrichment estimates across genes was 0.876. These strongly positive correlations of templates and enrichment estimates of marks known to co-occur suggest that co-regulatory information can be gleaned from spatial distribution and magnitude of the enrichment data (see Additional files 6 and 7 for all enrichment and template correlations). For example, our data show enrichment correlations of 0.889, 0.762, and 0.761 between H2BK5me1 and H3K79me1/2/3, respectively. Template correlations between H2BK5me1 and H3K79me1/2/3 were 0.956, 0.910, and 0.924, respectively. The high correlation between H2BK5me1 and H3K79 methylation deposition patterns and levels across the genome suggest that there may be a mechanistic link (e.g., the enzymes that deposit these marks could be on the same complex) between these histone PTMs that has not yet been reported in biochemical studies. This is one of many cases where both the correlation between two marks' spatial profiles and enrichment levels across genes is high (see Additional files 6 and 7 for all enrichment and template correlations). Using both enrichment level and spatial deposition patterns across genomes could prove to be powerful at identifying biologically relevant synergies between histone modifications, which make up the "histone code" [1-4].

## Conclusions

Generalizing a method for estimating ChIP-seq enrichment for multiple histone modifications is complicated by the variability in the way different modifications are deposited. This variability ultimately creates different gene-wise ChIP-seq enrichment patterns, some of which scale with gene length and some which do not. Tag counting methods can yield high quality predictors for regression modeling, but ultimately some of the information content coded in the spatial distribution of the data is lost. Although many modifications are highly enriched at the 5' end of genes, much of the useful data associated with a given gene is encoded in the body of the gene. Many previous studies have attempted to estimate enrichment by only focusing on the promoter region, and in doing so, have forgone much of the relevant data.

Using the MARS regression algorithm to build regression models with enrichment levels as predictors and gene expression as responses, we compared various strategies for estimating gene-wise ChIP-seq enrichment for 20 histone methylations and histone variant H2A.Z in human CD4^+ ^T cells [10]. Enrichment estimation methods were assessed and ranked by the quality of the models produced, which was measured by GCV scores. We have demonstrated that, with respect to the cis-regulatory role that the histone modifications/variant surveyed in this study play in controlling gene expression, the majority of the significant enrichment data lies within gene boundaries. Also, the incorporation of data across whole genes, as well as spatially weighting enrichment for single-value estimations of gene-wise ChIP-seq enrichment can provide significant improvement over strategies that focus on specific genic regions. Improving methods for the quantification of ChIP-seq data for statistical modeling serves to sharpen the resolution of the models and ultimately improves the conclusions that can be drawn from them.

ChIP-seq technology facilitates the computational interrogation of genomic control networks, and the conclusions drawn by this study can serve to increase depth at which we can probe these networks using this technology. The methods outlined in this work can be applied to almost any machine learning or data mining application that uses gene-wise ChIP-seq enrichment as predictors or responses.

## List of abbreviations

PTM: Posttranslational Modification; MARS: Multivariate Adaptive Regression Splines; GCV: Generalized Cross-Validation; CV(RMSD): Coefficient of Variation of the Root Mean Square Deviation.

## Competing interests

The authors declare that they have no competing interests.

## Authors' contributions

SAH calculated enrichment estimates, performed the MARS and CV(RMSD) analysis, drafted the manuscript and helped conceive the study. XX calculated correlations of enrichment estimates and templates, and helped draft the manuscript. SB helped conceive the study, draft the manuscript, and guided the analysis. All authors read and approved the final manuscript.

## Supplementary Material

Additional file 1 **Supplemental Table 1. Screening of exons for inclusion as an independent genic region for further analysis**. R^2 ^values for MARS models built with one mark as a predictor and gene expression as a response. The columns correspond to models that were built with tag counts from the Transcription Start Site (TSS), Transcription Termination Site (TTS), exons, or sums of two or more of the values. From this analysis we determined that the contributions of exons alone were relatively small, and would not be considered as an independent region for further analysis. Click here for file

Additional file 2 **Supplemental Figure 1. Example of a highly enriched 5' region on a large gene**. Enrichment of H3K4me3 and H2A.Z on ULK4 is highly 5' localized. Since ULK4 is over 700 kb in length, length-normalized enrichment estimates for these marks on this gene would be underestimated relative to most genes. Click here for file

Additional file 3 **Supplemental Figure 2. Histone modifications at locus where a 5' mark on one gene overlaps with the 3' region of another**. Five prime enrichment of H3K4me3 and H2A.Z on C12orf62 bleeds into the estimation window of GPD1, which is not enriched at its 5' end for either mark. A tag counting procedure would yield a large enrichment estimate of GPD1 relative to a template-based enrichment estimate since 3' enrichment is down-weighted for these marks using the template based procedure. Thus, for this and similar cases, the template-based enrichment estimates are better able to deconvolve neighboring ChIP-seq signals. Click here for file

Additional file 4 **Supplemental Figure 3. Average histone modification enrichments stratified by gene length**. Plots of average enrichment profiles from the transcription start site to 6000 bp into the gene body, stratified by quintiles of gene length. All marks are included except for H3K36me3, H3K79me2 and H3K79me3, which can be found in Figure 3. Most activating marks show decreased enrichment in longer genes, while repressive marks generally show increased enrichment in longer genes, suggesting decreased average gene expression in longer genes. Click here for file

Additional file 5 **Supplemental Figure 4. Gene expression stratified by gene length**. Box plots of gene expression stratified by quintiles of gene length. There is a significant decrease in expression in the longest 20%-ile of genes. Along with the observation that longer genes have relatively high enrichment of repressive marks, and low enrichment of activating marks, this suggests that lower gene expression in longer genes is mediated by epigenetic mechanisms. Click here for file

Additional file 6 **Supplemental Table 2. Spearman's rank correlation coefficients for all pairwise comparisons of enrichment estimates**. All pair-wise correlations between enrichment estimates calculated using the scaled-gene non-iterative estimation procedure with a 2000 bp intergenic overhang. Click here for file

Additional file 7 **Supplemental Table 3: Spearman's rank correlation coefficients for all pairwise comparisons of mark templates**. All pair-wise correlations between scaled-gene non-iterative templates with a 2000 bp intergenic overhang. Click here for file
