# Supplementary figures and images for: Quantification of histone modification ChIP-seq enrichment for data mining and machine learning applications
Source: BMC Res Notes. 2011 Aug 11;4:288. doi: 10.1186/1756-0500-4-288 (PMC3170335; doi:10.1186/1756-0500-4-288)

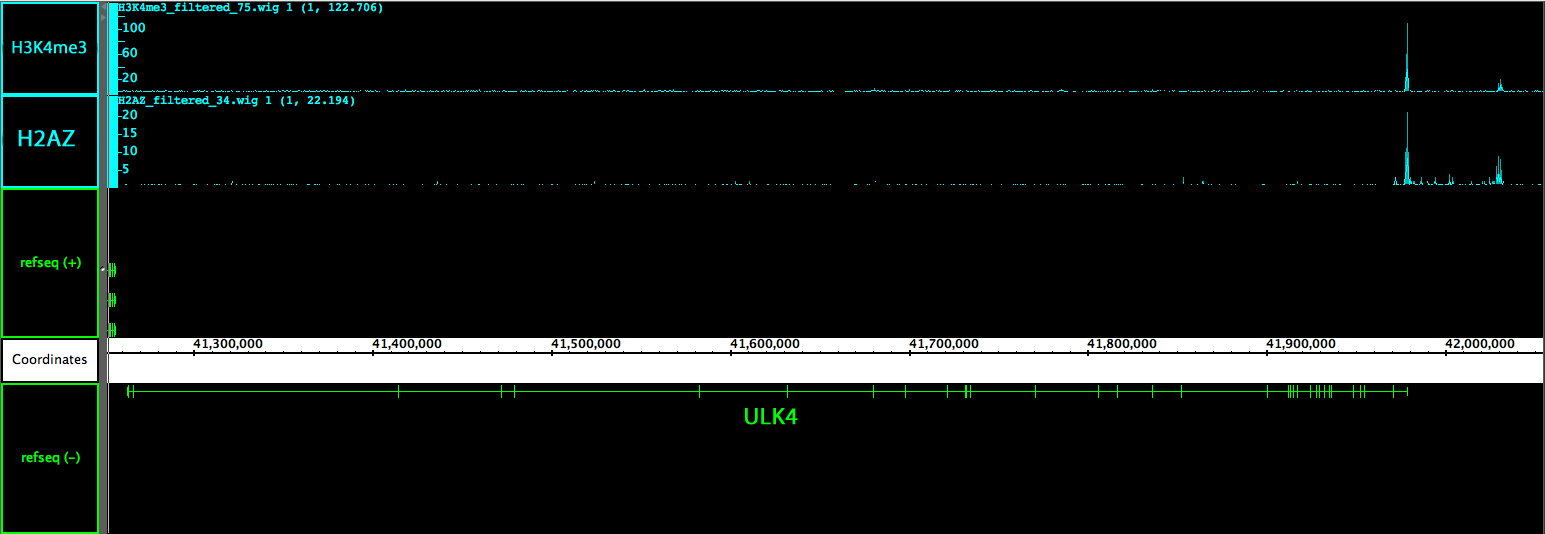

Supplement: Additional file 2 — Supplemental Figure 1. Example of a highly enriched 5' region on a large gene. Enrichment of H3K4me3 and H2A.Z on ULK4 is highly 5' localized. Since ULK4 is over 700 kb in length, length-normalized enrichment estimates for these marks on this gene would be underestimated relative to most genes. [file 1756-0500-4-288-S2.PNG]

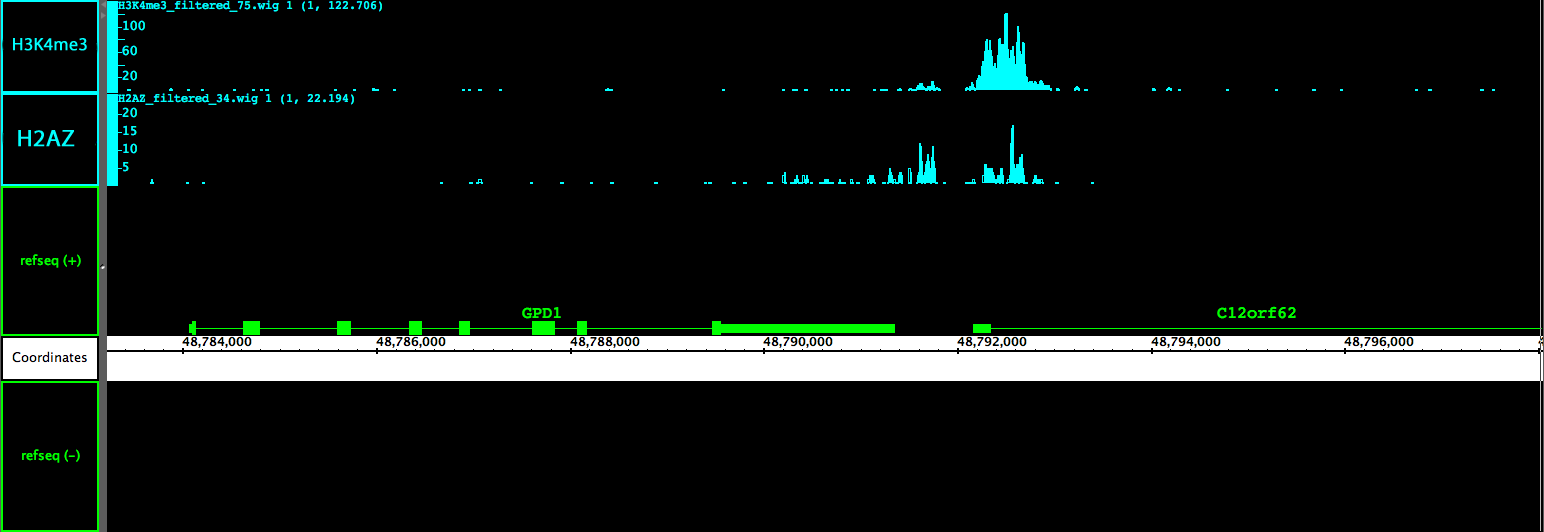

Supplement: Additional file 3 — Supplemental Figure 2. Histone modifications at locus where a 5' mark on one gene overlaps with the 3' region of another. Five prime enrichment of H3K4me3 and H2A.Z on C12orf62 bleeds into the estimation window of GPD1, which is not enriched at its 5' end for either mark. A tag counting procedure would yield a large enrichment estimate of GPD1 relative to a template-based enrichment estimate since 3' enrichment is down-weighted for these marks using the template based procedure. Thus, for this and similar cases, the template-based enrichment estimates are better able to deconvolve neighboring ChIP-seq signals. [file 1756-0500-4-288-S3.PNG]

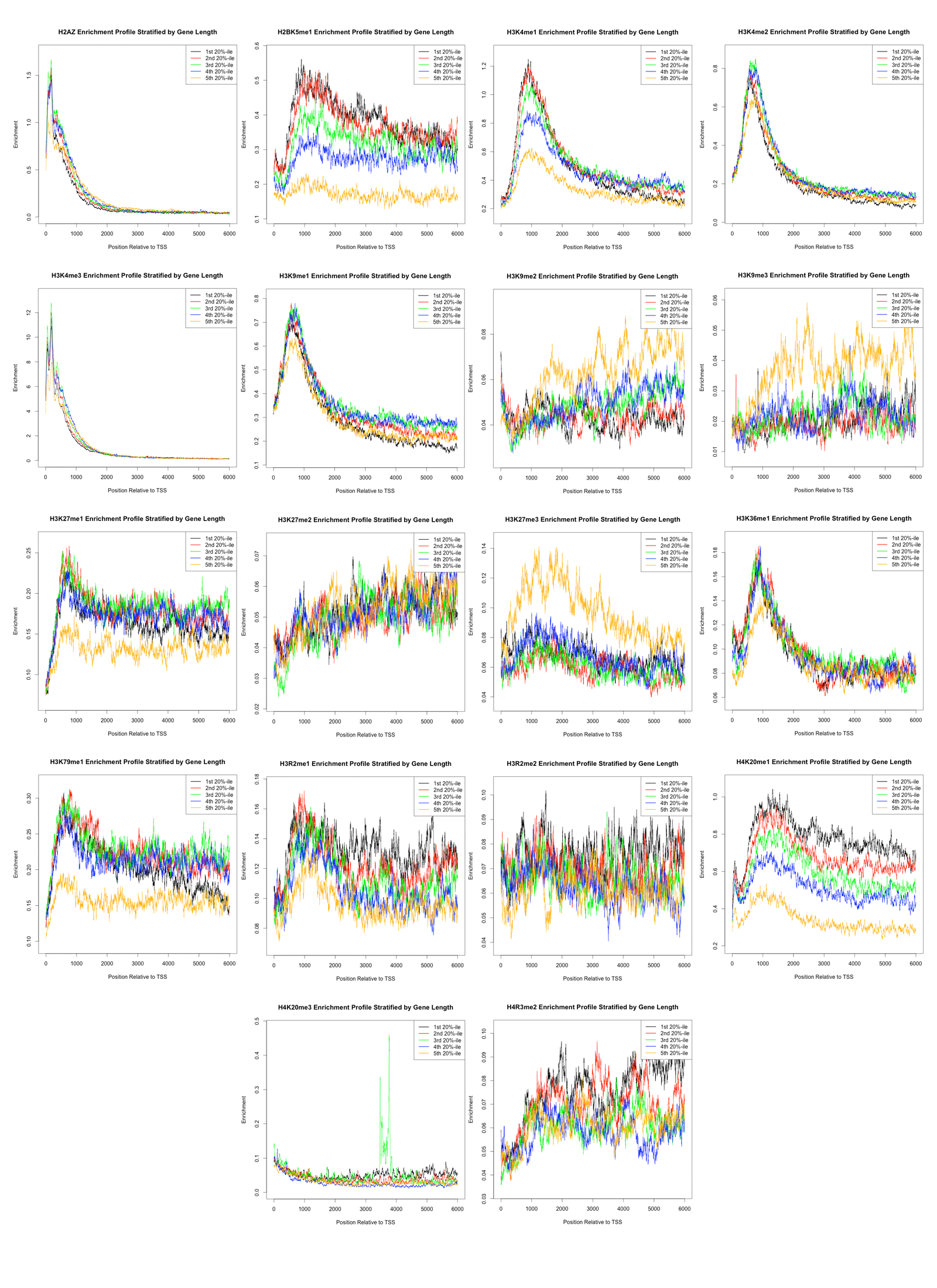

Supplement: Additional file 4 — Supplemental Figure 3. Average histone modification enrichments stratified by gene length. Plots of average enrichment profiles from the transcription start site to 6000 bp into the gene body, stratified by quintiles of gene length. All marks are included except for H3K36me3, H3K79me2 and H3K79me3, which can be found in Figure 3. Most activating marks show decreased enrichment in longer genes, while repressive marks generally show increased enrichment in longer genes, suggesting decreased average gene expression in longer genes. [file 1756-0500-4-288-S4.PNG]

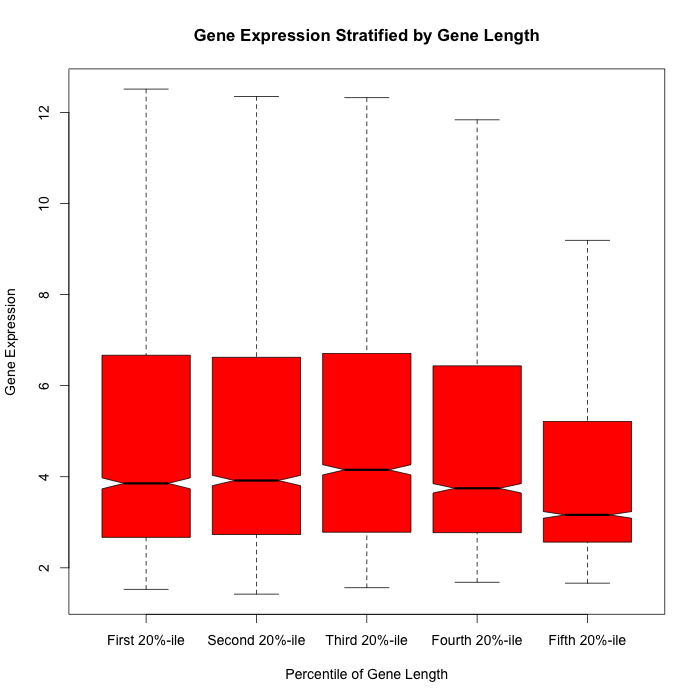

Supplement: Additional file 5 — Supplemental Figure 4. Gene expression stratified by gene length. Box plots of gene expression stratified by quintiles of gene length. There is a significant decrease in expression in the longest 20%-ile of genes. Along with the observation that longer genes have relatively high enrichment of repressive marks, and low enrichment of activating marks, this suggests that lower gene expression in longer genes is mediated by epigenetic mechanisms. [file 1756-0500-4-288-S5.PNG]
